# Utilization trends of pedicle subtraction osteotomies compared to posterior spinal fusion for deformity: a national database analysis between 2008–2011

**DOI:** 10.1186/s13013-016-0081-z

**Published:** 2016-08-24

**Authors:** Jeffrey L. Gum, Leah Y. Carreon, Jacob M. Buchowski, Lawrence G. Lenke, Steven D. Glassman

**Affiliations:** 1 Norton Leatherman Spine Center, 210 East Gray St. Suite 900, Louisville, KY 40202 USA; 2 Department of Orthopaedic Surgery, Washington University School of Medicine, 660 S Euclid Ave, Campus Box 8233, St. Louis, MO 63110 USA; 3 Columbia University Department of Orthopedic Surgery Division of Spinal Surgery, New York – Presbyterian Hospital, The Allen Pavilion, 5141 Broadway, New York, NY 10034 USA

**Keywords:** Pedicle subtraction osteotomy, Adult spine deformity, Spine deformity, Administrative claims dataset, Scoliosis, Kyphosis, Sagittal alignment

## Abstract

**Background:**

Increased awareness regarding the importance of the sagittal spinal profile has led to more aggressive correction of sagittal malalignment. The utilization trends of pedicle subtraction osteotomy (PSO) for sagittal plane correction in spinal deformity surgery have not been well characterized.

**Methods:**

A commercially available database (PearlDiver, Inc) was queried for both Private Payor and 5 % Medicare claims from 2008 to 2011. Revision and clarification of the coding guidelines for PSO were introduced in 2008. Patients who had a thoracic and/or lumbar PSO were identified using CPT codes (22206-22208). In order to appropriately interpret trends in PSO use, three comparison groups were identified. Patients who had a diagnosis of adult spine deformity were identified using ICD-9 codes. Patients who had fusion for spine deformity or posterior spine fusion were identified using CPT codes. Differences in annual utilization and demographics between these four groups were then compared.

**Results:**

From the Private Payor database, 199 PSOs were identified with the number of PSOs increasing from 33 in 2008, to 61 in 2011, representing a 185 % increase. From the Medicare data, 102 PSOs were identified, increasing from 13 in 2008 to 32 in 2011, a 246 % increase. In contrast, from both databases, there was minimal to no increase in the incidence of adult spine deformity, fusion for spine deformity or posterior spine fusion over the study time interval.

**Conclusion:**

Over the study time interval, there was up to a 3.2-fold increase in the utilization of PSOs while the diagnosis of adult spine deformity, fusion for spine deformity and posterior spine fusions had minimal to no increase.

## Background

The technique of pedicle subtraction osteotomy (PSO) was initially described by Thomasen et al. [1] in 1985 as a posteriorly based wedge-shaped osteotomy for kyphosis correction in patients with ankylosing spondylitis. Since then, the use of PSO has expanded, especially in the setting of adult spine deformity (ASD) correction. The last decade has brought an increased awareness of the importance of proper sagittal alignment of the spine. The sagittal vertical axis and spinopelvic parameters, such as lumbar lordosis and pelvic incidence are now known to be independent predictors of clinical outcomes after surgical correction of spine deformity [2–4]. The need to restore optimal sagittal alignment has lead surgeons to pursue more aggressive sagittal correction, for which PSO may be useful.

As PSO has become more widely accepted, even patients with flexible sagittal plane deformities have been considered candidates for PSO. However, in most instances flexible deformities can be managed appropriately with only posterior column osteotomies (PCOs) and typically, only rigid deformities require a PSO or 3-column osteotomy. With an aging population, adult spine deformity as well as fixed sagittal malalignment has become more commonly described in the literature [5–7]. We hypothesize there has been a marked increase in PSO utilization. Although Scheer et al. [8] suggest that procedures to address ASD have increased by 275 % over the last 10 years, the proportion of three-column osteotomies, specifically PSO, has yet to be evaluated. The purpose of this study is to characterize the utilization trends of PSO in comparison to the overall prevalence of ASD and the incidence of surgical treatment.

## Methods

A national insurance claims database (PearlDiver, Inc; Fort Wayne, IN) was queried for both Private Payor and 5 % Medicare claims from 2008 to 2011. The PearlDiver database is a public, commercially available Health Insurance Portability and Accountability Act–compliant national database compiled from a collection of private insurer records as well as a 5 % sampling of the Medicare claims database. The database has more than 2 billion individual patient records and contains Current Procedural Terminology (CPT) and International Classification of Diseases (ICD-9), Ninth Revision related to orthopedic procedures. From 2008 through 2011, the Private Payor database captured from 22.4 million to 26.3 million patients (8.3 to 9.9 % of the US population) in each year included in the analysis [9]. Thoracic and lumbar PSO procedures were identified using CPT codes (Table 1). Revision and clarification of the coding guidelines for a PSO were released in 2008 instructing physicians to report these procedures in addition to the arthrodesis. Therefore, 2008 was selected as the first time interval for the study. The selected PSO codes could potentially be utilized for thoracic and lumbar vertebral column resections. However, typically these procedures are coded using 63101-103 (vertebral corpectomy, vertebral body resection), partial or complete, lateral extracavitary approach with decompression of spinal cord and/or nerve root(s).

**Table 1:** Current Procedural Terminology (CPT) and International Classification of Diseases (ICD-9) used to identify cases

Pedicle subtraction osteotomy CPT codes	
22206	Osteotomy of spine, posterior or posterolateral approach, three columns, on vertebral segment (eg, pedicle/vertebral body subtraction), thoracic
22207	Osteotomy of spine, posterior or posterolateral approach, three columns, on vertebral segment (eg, pedicle/vertebral body subtraction), lumbar
22208	Osteotomy of spine, posterior or posterolateral approach, three columns, on vertebral segment (eg, pedicle/vertebral body subtraction), each additional level
Spine deformity ICD-9 codes	
73710	Kyphosis, acquired
73730	Scoliosis [and kyphoscoliosis], idiopathic
73734	Thoracogenic scoliosis
73739	Other kyphoscoliosis and scoliosis
73740	Spine curve unspecified
73741	Kyphosis associated with other conditions
73742	Lordosis associated with other conditions
73743	Scoliosis associated with other conditions
73850	Deformity, acquired, back/spine nec
Fusion for spine deformity CPT codes	
22800	Arthrodesis posterior for spinal deformity with or without cast; up to 6 vertebral segments
22802	Arthrodesis posterior for spinal deformity with or without cast; 7 to 12 vertebral segments
22804	Arthrodesis posterior for spinal deformity with or without cast; 13 or more vertebral segments
22808	Arthrodesis anterior for spinal deformity with or without cast; 2 to 3 vertebral segments
22810	Arthrodesis anterior for spinal deformity with or without cast; 4 to 7 vertebral segments
22812	Arthrodesis anterior for spinal deformity with or without cast; 8 or more vertebral segments
22610	Arthrodesis posterior or posterolateral technique single level; thoracic (with or without lateral transverse technique)
22612	Arthrodesis posterior or posterolateral technique single level; lumbar (with or without lateral transverse technique)
Posterior Spine Fusion CPT codes	
22610	Arthrodesis posterior or posterolateral technique single level; thoracic (with or without lateral transverse technique)
22612	Arthrodesis posterior or posterolateral technique single level; lumbar (with or without lateral transverse technique)

Since an increase in PSO utilization could be due several reasons, we wanted to provide a relative comparison to other spine surgeries during the same time interval. To make sure PSO utilization didn’t change because of a change in the overall number of spine surgeries being performed, we compared codes for spinal fusion. To make sure PSO utilization did not change because more patients are being diagnosed with ASD, we compared to ICD-9 diagnosis codes for ASD. As a third comparison, or relative control, we compared patients undergoing fusion for spinal deformity. In order to evaluate trends of PSO utilization in context, three comparison groups were identified. Patients with the diagnosis of ASD, patients undergoing fusion for spine deformity and patients undergoing posterior spine fusion (Table 1). The estimated number of procedures performed in the United States (US) was calculated from the incidence and US census data conversion factor. Demographic data, such as age, sex, and region, were also analyzed. Regions were defined as Midwest, Northeast, South, and West (Table 2).

**Table 2:** States grouped by region

Region	Sates
Midwest	IA, IL, IN, KS, MI, MN, MO, ND, NE, OH, SD, WI
Northeast	CT, MA, ME, NH, NJ, NY, PA, RI, VT
South	AL, AR, DC, DE, FL, GA, KY, LA, MD, MS, NC, OK, PR, SC, TN, TX, VA, WV
West	AK, AZ, CA, CO, HI, ID, MT, NM, NV, OR, UT, WA, WY

## Results

From the Private Payor database (Table 3), 199 total PSO cases were identified with the number of PSO’s increasing from 33 cases in 2008 to 61 cases in 2011 representing an 85 % increase over three years. The largest increase was in 2010 at 67 cases, an increase of 103 %. In contrast, the incidence of fusion for spine deformity decreased from 766 cases in 2008 to 743 cases in 2011. The number of posterior spine fusions increased slightly (4 %) from 8111 cases in 2008 to 8424 in 2011. The incidence of the diagnosis of ASD increased each year from 90,820 in 2008 to 99,099. The age ranges 55–59 and 60–64 each had 21 % (42/199) of the group with the number of PSO’s decreasing in each age range (50–54 = 31, 45–49 = 19, 40–44 = 16, 10–14 = 13). Forty-two percent (84/199) were done on patients between 55 and 64 years old and 67 % (134/199) were females. The South had the highest frequency at 76, followed by the West (65), Midwest (39), and Northeast (19).

**Table 3:** Number of patients undergoing pedicle subtraction osteotomy (PSO), fusion for spine deformity and posterior spine fusion who were diagnosed with adult spine deformity by year in the United States (US) Private Insurance Database (Medicare not included) represented in the PearlDiver Database

Year	Number of procedures				Patients in database	US estimates			
	PSO	Fusion for deformity	Posterior spine fusion	Adult spine deformity		PSO	Fusion for deformity	Posterior spine fusion	Adult spine deformity
2008	33	766	8,111	90,820	26,345,000	346	8,030	85,031	952,100
2009	38	750	8,172	91,686	24,625,000	430	8,496	92,572	1,038,620
2010	67	795	8626	93,537	24,810,000	756	8,975	97,386	1,056,018
2011	61	734	8,424	99,099	25,870,000	661	7,959	91,348	1,074,609
Total	199	3,045	33,333	375,142	198,876,000	2,178	33,327	364,826	4,105,885

From the Medicare claims database (Table 4), a total of 102 PSO cases were identified. There was an increase from 13 in 2008 to 32 in 2011. This is an increase of 146 %. Similar to the Private Payor database, the largest increase occurred in 2010 at 42 cases, an increase of 223 %. The incidence of fusion for spine deformity increased by 11 %, from 101 in 2008 to 112 in 2011. The number of posterior spine fusions increased by 26 % from 2882 in 2008 to 3624 in 2011. The incidence of the diagnosis of ASD increased each year from 28,172 in 2008 to 33,376. Thirty-seven percent (37/102) were performed in patients under the age of 65 years old (65–69 = 27, 70–74 = 20, 75–79 = 13) and 66 % (66/102) were females. The South, again, had the highest frequency at 40, followed by the West (34), Midwest (15), and (10) Northeast (11).

**Table 4:** Number of patients undergoing pedicle subtraction osteotomy (PSO), fusion for spine deformity and posterior spine fusion who were diagnosed with adult spine deformity by Year in the United States (US) from the 5 % Medicare sample represented in the PearlDiver Database

Year	Number of procedures				US estimates			
	PSO	Fusion for deformity	Posterior spine fusion	Adult spine deformity	PSO	Fusion for deformity	Posterior spine fusion	Adult spine deformity
2008	13	101	2,882	28,172	260	2,020	57,640	563,440
2009	15	87	3,293	29,841	300	1,740	65,860	596,820
2010	42	99	3,540	30,668	840	1,980	70,800	613,360
2011	32	112	3,624	33,376	640	2,240	72,480	667,520
Total	102	399	13,339	122,057	2,040	7,980	266,780	2,441,140

## Discussion

Adult spine deformity (ASD) is an inevitable consequence of our ever-aging population, and therefore looms as a major healthcare issue in the 21st century [10]. In an effort to appropriately address the complex and challenging nature of surgical ASD management, great attention has been placed on optimizing improvement in health-related quality of life outcomes. Although ASD is comprised of many types of pathology, sagittal malalignment, in particular, has been shown to be a significant driver of poor clinical outcomes [3]. The necessity of sagittal realignment or restoration in ASD typically requires a complex procedure, often including a long posterior fusion and spinal osteotomies [8, 11]. Guidance regarding correction thresholds is emerging, and can be very useful in preoperative planning for these complex procedures [12]. Enthusiasm for this data-driven trend toward aggressive realignment of sagittal plane parameters has popularized the use of three-column osteotomies, specifically pedicle subtraction osteotomy.

As surgeon experience and techniques are evolving, the complication rate, although still high, appears to be declining. Auerbach et al. [13] reported a retrospective review of 87 PSOs with a complication rate of 38 % which is less than Cho et al.’s [14] series highlighting a 58 % complication rate. Although the overall complication rate may be more acceptable, an increase in the frequency of proximal junctional kyphosis (PJK) is evident [15]. Not only is PJK becoming more significant, the catastrophic counterpart, proximal junctional failure (PJF) which requires revision surgery, has been increasingly recognized. Dramatic improvement in sagittal alignment or lumbar lordosis correction seen with PSO has been directly correlated with increased risk for PJF [15]. Additionally, although PSO was initially described as a treatment of a fixed deformity, the distinction between a flexible deformity and fixed deformity is not concrete and has yet to be clarified in the current literature. The distinction between flexible and fixed ASD malalignment should be a future goal in the ASD literature.

In order to refine indications and minimize complications it is important to monitor utilization trends of complex procedures, such as PSOs. To our knowledge, no study has examined the utilization of PSO. We sought to characterize the utilization trends of PSO with respect to three comparison groups representative of ASD. Over the study time interval, there was a substantial increase in the utilization of PSOs in both the Private Payor and Medicare populations, 85 and 146 % respectively. The maximal increase relative to 2008 for both populations was in 2011 with a 2.0- and 3.2-fold increase (103 and 223 %). The greatest increase for the two fusion comparison groups from the Private Payor population was also in 2011, but this increase was minimal. The diagnosis of ASD and the incidence surgical treatment increased each year. Although, not a primary goal of this study, the results showed that ASD as a diagnosis is increasing over the study time interval (9–26 %). This emphasizes the importance of optimizing care for this population, as this trend will likely continue with our aging population.

The study has several limitations including the retrospective nature and the potential for inconsistencies in the manner a PSO is coded. Although likely not an issue, the number of PSO procedures is relatively low as compared to the other study groups. Therefore, a small number of miscoded PSO procedures could significantly alter the results. Especially in the Medicare data, as this is a 5 % sample that is representative of the US population. As mentioned in the methods, changes in coding guidelines were released in 2008, which is the first year included in the study. The increase in utilization could partly be due to increasing awareness of these guidelines. Ideally, we would have a longer study time interval and several years after any new coding guidelines.

## Conclusions

The number of PSO has increased dramatically when compared to related groups consisting of spine fusion for ASD, posterior spine fusion, and ASD as a diagnosis. Although PSO is a powerful technique to improve sagittal malalignment in ASD patients, it is complex and carries a high complication rate. It is important that as we optimize clinical outcomes and the delivery of spine care, we optimize the utilization of complex procedures with well-defined indications.
